# Differential expression of *CXCR1* and commonly used reference genes in bovine milk somatic cells following experimental intramammary challenge

**DOI:** 10.1186/s12863-015-0197-9

**Published:** 2015-04-22

**Authors:** Joren Verbeke, Mario Van Poucke, Luc Peelman, Sarne De Vliegher

**Affiliations:** M-team and Mastitis and Milk Quality Research Unit, Department of Reproduction, Obstetrics and Herd Health, Faculty of Veterinary Medicine, Ghent University, Salisburylaan 133, Merelbeke, Belgium; Animal Genetics Laboratory, Department of Nutrition, Genetics, and Ethology, Faculty of Veterinary Medicine, Ghent University, Heidestraat 19, Merelbeke, Belgium

**Keywords:** *CXCR1*, RT-qPCR, Milk somatic cell, Experimental intramammary challenge

## Abstract

**Background:**

Chemokine (C-X-C motif) receptor 1 (CXCR1 or IL-8RA) plays an important role in the bovine mammary gland immunity. Previous research indicated polymorphism c.980A > G in the *CXCR1* gene to influence milk neutrophils and mastitis resistance. In the present study, four c.980AG heifers and four c.980GG heifers were experimentally infected with *Staphylococcus chromogenes*. RNA was isolated from milk somatic cells one hour before and 12 hours after the experimental intramammary challenge. Expression of *CXCR1* and eight candidate reference genes (*ACTB*, *B2M*, *H2A*, *HPRT1*, *RPS15A*, *SDHA*, *UBC* and *YWHAZ*) was measured by reverse transcription quantitative real-time PCR (RT-qPCR). Differences in relative *CXCR1* expression between c.980AG heifers and c.980GG heifers were studied and the effect of the experimental intramammary challenge on relative expression of *CXCR1* and the candidate reference genes was analyzed.

**Results:**

Relative expression of *CXCR1* was not associated with polymorphism c.980A > G but was significantly upregulated following the experimental intramammary challenge. Additionally, differential expression was detected for *B2M*, *H2A*, *HPRT1*, *SDHA* and *YWHAZ*.

**Conclusions:**

This study reinforces the importance of *CXCR1* in mammary gland immunity and demonstrates the potential effect of experimental intramammary challenge on expression of candidate reference genes in milk somatic cells.

## Background

After invading the bovine mammary gland, pathogens can cause an intramammary infection (IMI) followed by an inflammatory response called mastitis. Neutrophils migrating from blood to milk play an important role in the mammary gland immunity [1]. Binding of cytokine interleukin-8 on chemokine (C-X-C motif) receptor 1 (CXCR1) causes chemotaxis and enhances viability of bovine neutrophils [2,3]. Despite its important function, many single nucleotide polymorphisms (SNP) were detected in the *CXCR1* gene [4,5]. Recently, we reported a higher milk neutrophil viability and lower likelihood of IMI by major mastitis pathogens (e.g. *Staphylococcus aureus* and *Streptococcus uberis*) in heifers with genotype *CXCR1*c.980AG compared to heifers with genotype *CXCR1*c.980GG [4,6]. Polymorphism c.980A > G causes the amino acid change p.Lys327Arg in the C-terminal region of the receptor potentially influencing interleukin 8 signal transduction. However, phenotypical differences could also be explained by linkage with SNPs in regulatory regions and an association between SNP c.980A > G and *CXCR1* gene expression. To test this hypothesis, we isolated RNA from milk somatic cells isolated before and after an experimental challenge with *Staphylococcus chromogenes*. Next, differences in *CXCR1* expression between c.980AG and c.980GG heifers were studied using reverse transcription quantitative real-time PCR (RT-qPCR). Additionally, the influence of the experimental intramammary challenge on expression of *CXCR1* and commonly used reference genes was analyzed.

## Methods

### Test animals

This experiment has been approved by the ethical committee of the Faculty of Veterinary Medicine, Ghent University (EC2012/73). A blood sample was taken from all Holstein heifers (n = 20) of the commercial dairy herd of Ghent University (Biocenter Agri-Vet, Melle, Belgium). The whole coding region of *CXCR1* was genotyped by direct sequencing as previously described [4]. Four heifers with genotype c.980AG and 4 heifers with genotype c.980GG were selected. Selected heifers were not siblings, had no history of clinical mastitis or other diseases, and were between 75 and 280 days in milk at the time of the experiment. Duplicate milk samples were taken four days and one hour before the experiment. Bacteriological culture was performed according to National Mastitis Council (NMC) guidelines [7]. All quarters of all heifers were culture-negative at that time. Quarter SCC was measured before and during the experiment in duplicate using a DeLaval cell counter (DCC, DeLaval International AB, Tumba, Sweden).

### Experimental challenge

**Figure 1:**
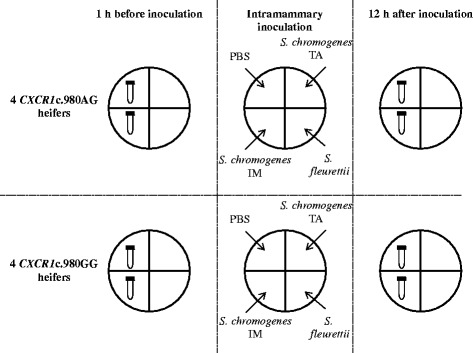
Samples. Four *CXCR1*c.980AG heifers and four *CXCR1*c.980AG heifers were inoculated with PBS, a *Staphylococcus chromogenes* strain isolated from a chronic intramammary infection (*S. chromogenes* IM), a *Staphylococcus chromogenes* strain from a teat apex (*S. chromogenes* TA) and a *Staphylococcus fleurettii* strain in a split-udder design to study differences between coagulase-negative staphylococci. For this research, somatic cells were isolated from milk samples taken 1 h before and 12 h after inoculation from quarters (to be) inoculated with PBS or *S. chromogenes* IM.

Data were available from a larger experimental infection study in which each heifer was inoculated briefly after the morning milking (8 a.m.) with two different strains of *S. chromogenes*, a strain of *Staphylococcus fleurettii*, and sterile phosphate buffered saline (PBS) in a split-udder design (one strain or PBS per individual quarter) to study differences in pathogenicity and immune response between coagulase-negative staphylococci. One *S. chromogenes* (*S. chromogenes* IM) strain originated from a chronically infected quarter [8] whereas the other (*S. chromogenes* TA) originated from the teat apex and was found to inhibit the growth of major pathogens *in vitro* [9]. The *S. fleurettii* strain originated from sawdust [8]. For each strain, 1*10^6^ CFU in 5 mL PBS was inoculated using a sterile catheter (Vygon, Ecouen, France). The bacterial count was determined by incubating a tenfold serial dilution of a representative frozen aliquot 18 h before inoculation. Five mL of sterile PBS was inoculated in the fourth quarter (further referred to as neighboring quarters). For this research, additional milk samples of 600 mL were taken 1 h before and 12 h after inoculation from quarters (to be) inoculated with PBS or *S. chromogenes* IM (Figure 1). Cows were milked after sampling.

### Milk somatic cell isolation

Samples were transported on ice to the laboratory where milk was divided equally between three 400-mL centrifuge bottles, diluted 50% (vol/vol) with cold PBS, and centrifuged at 1500 × g for 15 min at 4°C in a fixed angle rotor. The supernatant was discarded. The three milk somatic cell pellets were resuspended in a total of 40 mL PBS, divided between two 50-mL Falcon tubes and washed three times with 10 mL cold PBS (centrifugation at 200 x g for 10 min at 4°C). The final milk somatic cell pellets were suspended in 1 mL of RPMI 1640 (Gibco Brl., Scotland, UK) supplemented with 1% BSA (Merck KGaA, Darmstadt, Germany). Twenty μL of the suspension was diluted with 380 μl low SCC milk (SCC < 50 cells/mL) and measured with a DeLaval cell counter to estimate the cell concentration using following formula; SCCsample (in cells/μl) $=\frac{400*{\mathrm{SCC}}_{\mathrm{mix}}-380*{\mathrm{SCC}}_{\mathrm{milk}}}{20}$. Approximately 5*10^6^ cells were pipetted in a 2 mL test tube, pelleted by centrifugation at 16,100 x g for 1 min at 4°C and resuspended in 1 mL TRI Reagent Solution (Ambion, Austin, TX). If less cells were available, all of the cell suspension was used. Samples were frozen and stored at -20°C for 8-10 months.

### RNA extraction and cDNA synthesis

RNA was isolated following the manufacturer’s instructions of TRI Reagent Solution (Ambion, Austin, TX). Genomic DNA was removed by adding 4 μL RQ1 DNase (0.5 U/μL, Promega, Leiden, Netherlands) and 2.7 μL RQ1 DNase 10X Reaction Buffer (Promega) followed by incubation for 30 min at 37°C. The reaction was terminated by adding 3 μL RQ1 DNase Stop Solution (Promega) followed by incubation for 10 min at 65°C. The RNA was purified by spin-column centrifugation (Amicon Ultra-0.5 centrifugal filter device, Merck Millipore, Billerica, MA) to approximately 15 μL. Its concentration and purity was estimated using a ND-1000 spectrophotometer (NanoDrop, Wilmington, NC). RNA degradation was analyzed by gel electrophoresis of a representative sample. Due to low yield in many samples, an additional PCR assay was designed to analyze cDNA integrity of all samples (see further).

DNA contamination was assessed by performing a minus RT control. A PCR mix, in a total volume of 10 μL, containing 0.4 μL sample (double the equivalent of RNA input in the qPCR reaction), 1 μL 10 × FastStart Taq DNA Polymerase Buffer (Roche Applied Science), 0.2 μl dNTP Mix (10 mM each; BIOLINE, London, UK), 0.5 μL forward primer [*YWHAZ* + 1, 5 μM, 5’-GCATCCCACAGACTATTTCC-3', IDT (Integrated DNA Technologies), Leuven, Belgium], 0.5 μL reverse primer (*YWHAZ*-1, 5 μM, 5’-GCAAAGACAATGACAGACCA-3', IDT) and 0.1 μL Taq DNA Polymerase (5 U/μl, Roche Applied Science) was made. The PCR program consisted of an initiation step of 4 min at 95°C followed by 40 amplification cycles (denaturation for 10 s at 95°C, annealing for 10 s at 58°C and extension for 20 s at 72°C) and a final 2-min elongation step at 72°C. DNA amplification (120 bp) was examined by electrophoresis on ethidium bromide-stained agarose (2%) gel (150 V, 20 min).

Improm-II reverse transcriptase (Promega) was used to convert DNA-free RNA into cDNA. First, 10 μL sample containing a maximum of 1 μg RNA was mixed with 0.8 μL random hexamer primers (10 μM, IDT) and 0.8 μL oligo(dT)_15_ primer (10 μM, IDT). If less than 1 μg RNA was extracted, all available RNA was used. The primer/template mix was thermally denatured by 5 min incubation at 70°C followed by 5 min incubation on ice. Secondly, 4 μL Improm-II 5x reaction buffer, 2.4 μL MgCl_2_ (25 mM), 1 μL dNTP Mix (10 mM each; BIOLINE) and 1 μL Improm-II reverse transcriptase (20 U/μL) was added. The final volume of 20 μL was incubated 5 min at 25°C (primer annealing), 60 min at 42°C (first-strand synthesis reaction), and 15 min at 70°C (inactivation of reverse transcriptase). Samples were 5 times diluted and stored at -20°C.

### PCR assay to assess cDNA integrity

**Figure 2:**
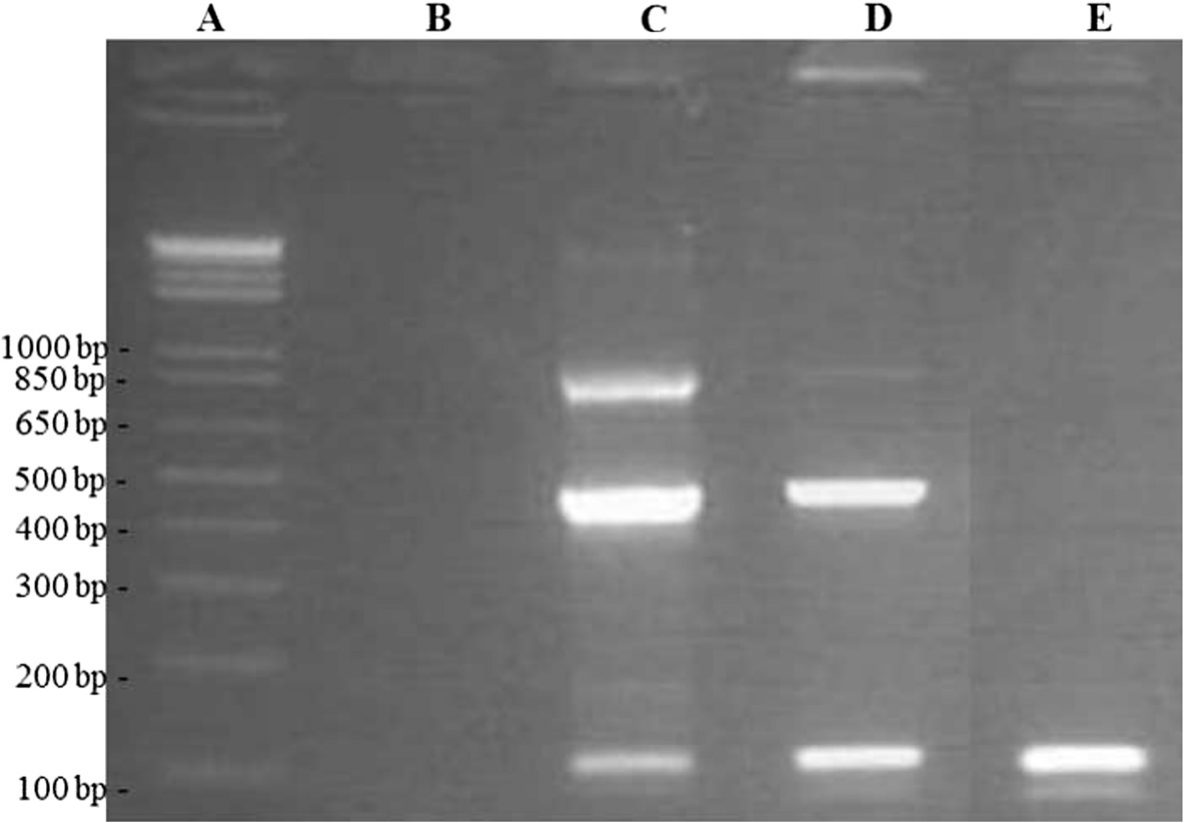
Agarose gel of a PCR assay with 4 *YWHAZ* primer used to assess cDNA integrity. Lane **A)** Marker (1-kb + DNA Ladder, Promega, Madison, WI); lane **B)** No template control; lane **C)** cDNA sample with amplified fragments of approximately 100, 500 and 900 bps (excellent integrity); lane **D)** cDNA sample with amplified fragments of approximately 100 and 900 bps (sufficient integrity) and lane **E)** cDNA sample with amplified fragment of approximately 100 bps (insufficient integrity).

Complementary DNA integrity was assessed using two 4-primer PCR assays multiplying fragments of approximately 100, 500 and 900 bps of *YWHAZ* and *CXCR1*, respectively. For both assays, a forward and 3 reverse PCR primers were designed using Primer3Plus [10] and synthesized by IDT. Sequences are shown in Table 1. Regions forming potential secondary structures were identified with Mfold [11] and avoided. Specificity of binding of the primers was analyzed using NCBI BLAST [12]. For both assays, a PCR mix, in a total volume of 10 μL, containing 2 μL 5x diluted cDNA, 1.0 μL 10 × FastStart Taq DNA Polymerase Buffer (Roche Applied Science), 0.3 μL dNTP Mix (10 mM each; BIOLINE), 1 μL forward primer (5 μM), 0.3 μL reverse primer 1 (5 μM), 0.3 μL reverse primer 2 (5 μM), 0.6 μL reverse primer 3 (5 μM) and 0.1 μL Taq DNA Polymerase (5 U/μl, Roche Applied Science) was made. The PCR program consisted of an initiation step of 4 min at 95°C followed by 40 amplification cycles (denaturation for 45 s at 95°C, annealing for 45 s at the optimal annealing temperature and extension for 1 min 30 s at 72°C) and a final 4-min elongation step at 72°C. The optimal annealing temperature for the *YWHAZ* and *CXCR1* assay (60°C and 65°C, respectively) were determined experimentally. Complementary DNA amplification was examined by electrophoresis on ethidium bromide-stained agarose (0.8%) gel (150 V, 25 min). The cDNA integrity was considered excellent, sufficient or insufficient if, respectively, 3, 2 or 1 bands were visible in both assays (Figure 2).

**Table 1:** **Primers used in two PCR assays multiplying three fragments of** ***YWHAZ*** **and** ***CXCR1*** **, respectively**

**Gene**	**Primer**	**Sequence (5’ → 3’)**	**Product size (bp)**
*YWHAZ* ^*a*^	Forward	GAGCAAAAGACGGAAGGTGCT	
	Reverse 1	CCAAAAGAGACAGTACATCATTGCA	109
	Reverse 2	TCCGATGTCCACAATGTCAAGT	497
	Reverse 3	TCCCCACCAGGACATACCAA	909
*CXCR1* ^*b*^	Forward	TCCCTGTGAGATAAGCACTGAGACA	
	Reverse 1	AGCGACCAATCCGGCTGTA	131
	Reverse 2	CGTTGTATTGGCACCCAGGTC	505
	Reverse 3	GTCCGTGGCGAAACTTCTGG	863

^a^Tyrosine 3-monooxygenase/tryptophan 5-monooxygenase activation protein, zeta polypeptide [NM_174814.2].

^b^Chemokine (C-X-C motif) receptor 1 [NM_174360.2].

### RT-qPCR

Ten candidate reference genes were selected based upon previous research [13-15]: *ACTB*, *B2M*, *H2A*, *HPRT1*, *PPP1R11*, *RPS15A*, *SDHA*, *TBP*, *UBC*, and *YWHAZ*. Primers were ordered from IDT. Gene, primer and amplicon information is listed in Table 2. A PCR mix of 10 μL containing 5 μL 2x SYBR Green I Master Mix (Roche Diagnostics, Basel, Switzerland), 1 μL forward primer (5 μM), 1 μL reverse primer (5 μM) and 2 μL cDNA sample was made. The PCR program consisted of an initiation step of 3 min at 95°C, followed by 40 amplification cycles (denaturation for 30 s at 95°C, annealing-elongation for 40 s at the optimal annealing temperature and detection of fluorescent signals generated by SYBR Green I binding to dsDNA). Samples were heated from 75°C to 95°C in 0.5°C increments per 5 s while continuously measuring fluorescence. The generated melt curve was used to confirm a single gene-specific peak and to detect primer/dimer formation. Optimal annealing temperatures were determined experimentally by gradient qPCR on a 4-fold serial dilution until 1/1024 of pooled cDNA of all samples. All reactions were performed in duplicate. In each run, the serial dilution and a no template control were included to analyze calibration curves, PCR efficiency (E) and squared correlation coefficient (r^2^) and check for PCR contamination. All qPCRs were performed in PCR strips (Bio-Rad, Hercules, CA) using a CFX96 Touch™ Real-Time PCR Detection System (Bio-Rad). Quantification cycles (Cqs) were analyzed with CFX Manager™ Software v3.1 (Bio-Rad). The raw Cq values were converted to quantiles (Q) using following formula; Q = (1 + E)^(CqS – CqL)^ with E = PCR efficiency, CqS = Cq value of the sample and CqL = lowest Cq value of all samples.

**Table 2:** **Gene, primer and amplicon information**

**Gene full name**	**NCBI Gene ID**	**Genbank Accession number**		**Sequence (5’ → 3’)**	**Genomic location**	**Product size (bp)**	**Ta (°C)**
*ACTB*	280979	NM_173979.3	F^a^	CCTCACGGAACGTGGTTACA	CDS^b^	87	58
Actin, beta			R^a^	TCCTTGATGTCACGCACAATTT	CDS		
*B2M*	280729	NM_173893.3	F	AGACACCCACCAGAAGATGG	CDS	206	59
Beta-2-microglobulin			R	CGGCAGCTGTACTGATCCTT	CDS		
*CXCR1*	281863	NM_174360.2	F	TCCCTGTGAGATAAGCACTGAGACAC	CDS	118	64
Chemokine (C-X-C motif) receptor 1			R	GCTGTATAAGATGACCAGCATCACCA	CDS		
*H2A*	506900	NM_001205596.1	F	GTCGTGGCAAGCAAGGAG	CDS	182	60
Histone 2 alpha			R	GATCTCGGCCGTTAGGTACTC	CDS		
*HPRT1*	281229	NM_001034035.2	F	TGCTGAGGATTTGGAGAAGG	CDS	154	58
Hypoxanthine phosphoribosyl-transferase I			R	CAACAGGTCGGCAAAGAACT	CDS		
*PPP1R11* ^*4*^	504846	NM_001100295.1	F	ACCATCAAACTTCGGAAACG	CDS	166	57
Protein phosphatase 1, regulatory (inhibitor) subunit 11			R	CCTCCTCTTCCTCGTCATCA	CDS		
*RPS15A*	337888	NM_001037443.2	F	AATGTCCTGGCTGATGCTCT	CDS	218	59
Ribosomal protein S15a			R	GGGCTGATCACTCCACACTT	CDS		
*SDHA*	281480	NM_174178.2	F	GCAGAACCTGATGCTTTGTG	CDS	185	60
Succinate dehydrogenase flavoprotein subunit A			R	CGTAGGAGAGCGTGTGCTT	CDS		
*TBP* ^*5*^	516578	NM_001075742.1	F	AATGGCTGCTGTGTTCTCCT	3’UTR^c^	214	60
TATA box binding protein			R	TGACGCTCTGGTGTTCTCTGT	3’UTR		
*UBC*	444874	NM_001206307.1	F	AGTTCAGTCTTCGTTCTTCTGTG	5’UTR^d^	88	58
Ubiquitin C			R	GGTTTTACCAGTGAGGGTCTT	CDS		
*YWHAZ*	287022	NM_174814.2	F	GCATCCCACAGACTATTTCC	CDS	120	60
Tyrosine 3-monooxygenase/tryptophan 5-monooxygenase activation protein, zeta polypeptide			R	GCAAAGACAATGACAGACCA	CDS		

^a^Forward and reverse primer.

^b^Coding sequence.

^c^3’ untranslated region.

^d^5’ untranslated region.

### Analysis of gene expression stability

Stability of the different candidate reference genes were analyzed using Normfinder (version 0.953, [16]) Excel Add-In. Samples were grouped as (1) all samples 1 h before inoculation, (2) samples from quarters inoculated with PBS 12 h after inoculation, and (3) samples from quarters inoculated with *S. chromogenes* IM 12 h after inoculation. Normfinder estimates an expression stability measure (ρ) per candidate reference gene based on the overall variation of the expression and the variation of the expression between the subgroups [16].

### Data analysis

Differences in gene expression between *CXCR1* genotype and sample subgroups were further studied using SAS 9.4 (SAS Institute Inc., NC, USA). First, expression of *B2M, CXCR1*, *H2A*, *HPRT1*, *SDHA* and *YWHAZ* were normalized by the geometric mean of the three most stable genes (*ACTB*, *RPS15A* and *UBC*; see further). Expression of *ACTB*, *RPS15A* and *UBC* were normalized to the geometric mean of the other two most stable genes. Data were log transformed to obtain a normalized distribution. Secondly, a linear mixed regression model was fit with relative expression as outcome variable and heifer and quarter as random effect to correct for clustering of quarters within cows and two observations per quarter, respectively (PROC MIXED, SAS 9.4). Sample subgroup (1, 2 and 3) was added as fixed effect. In the model for *CXCR1,* genotype (c.980AG and c.980GG) and the interaction between sample subgroup and genotype were also tested. Statistical significance was assessed at *P* ≤ 0.05.

## Results

### Experimental challenge

All milk samples taken 24, 12 and 1 h before experimental inoculation were culture negative. Quarters (n = 16) had a geometric mean SCC of 48,000 cells/ml [Interquartile range (IQR) 26,000 – 84,000] 1 h before inoculation. Neighboring quarters remained culture negative after inoculation. Quarters inoculated with the *S. chromogenes* IM developed subclinical mastitis as no local or systemic signs were observed and the challenge isolate was recovered from all quarters 6 h after inoculation. Twelve hours after inoculation, neighboring and infected quarters had a geometric mean SCC of 77,000 cells/ml (IQR 44,000 to 126,000) and 1,692,000 cells/ml (IQR 861,000 to 3,371,000), respectively.

### Quality control of nucleic acids

Total amount of isolated RNA ranged from 27.6 ng to 36.5 μg per sample. An average A_260_/A_280_ ratio of 2.04 was measured (range: 1.81-2.23). Gel electrophoresis of a representative sample indicated high quality rRNA. Additionally, the cDNA integrity was assessed using two four-primer PCR assays amplifying three different *YWHAZ* fragments and three different *CXCR1* fragments*.* In two samples, gel electrophoresis of both assays showed no or only one band indicating cDNA integrity to be too low to amplify the medium and large fragments. Latter samples were not further analyzed. The cDNA integrity of the remaining samples was considered sufficient (2 bands, n = 8) or excellent (3 bands, n = 22). Results of the *YWHAZ* and *CXCR1* assay were concordant.

### RT-qPCR and gene expression stability

**Figure 3:**
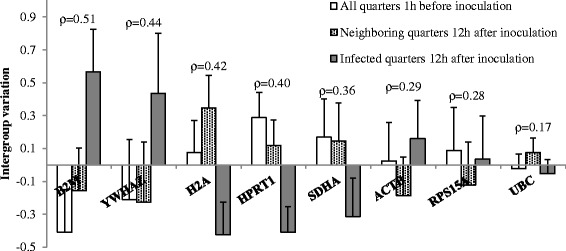
Gene expression stability of candidate reference genes in milk somatic cells analysed using Normfinder software. Intergroup variation (+ intragroup variation) of expression of 8 candidate reference genes in milk somatic cells isolated from quarters inoculated with PBS (n = 8) or *Staphylococcus chromogenes* (n = 8) of 8 dairy heifers. Candidate reference genes are ranked on gene expression stability (ρ) calculated using Normfinder [16] with most stable genes on the right side (smallest inter- and intragroup variation).

The gradient qPCR of *PPP1R11* and *TBP* on the 1/1024 dilution of the pooled cDNA showed weak fluorescent signals and melt peaks indicating low expression of these genes in the samples. They were not further tested as reference genes. The calibration curves of the remaining candidate reference genes and *CXCR1* demonstrated PCR efficiencies close to 100% and correlation coefficients close to 1 indicating good assay performance. Median Cq values were low. The SD of the Cq values of replicate samples were limited demonstrating good repeatability (Table 3). Normfinder identified *UBC*, *RPS15A* and *ACTB* as the most stable genes based on their low inter- and intragroup variation in expression (Figure 3).

**Table 3:** **qPCR information**

**Gene**	**Median Cq** ^**a**^	**SD Cq** ^**b**^	**Calibration curve** ^**c**^			
			**slope**	**y intercept**	**E (%)**	**r** ^**2**^
*ACTB*	22.5	0.125	-3.541	18.607	91.6	0.997
*B2M*	17.7	0.137	-3.426	13.756	95.8	0.998
*CXCR1*	24.3	0.077	-3.195	17.240	105.6	0.999
*H2A*	23.1	0.186	-3.370	19.463	98.0	0.996
*HPRT1*	25.4	0.140	-3.463	20.475	94.4	0.997
*RPS15A*	19.3	0.151	-3.433	15.213	95.6	0.999
*SDHA*	23.4	0.148	-3.534	19.385	91.8	0.999
*UBC*	22.5	0.122	-3.248	18.150	103.2	0.998
*YWHAZ*	22.8	0.097	-3.368	19.139	98.1	0.999

^a^Median of the quantification cycle (Cq) of all samples.

^b^Average standard deviation of the Cq values of replicate samples.

^c^Slope, y intercept, PCR efficiency and correlation coefficient estimated by qPCR on a 4-fold serial dilution of pooled cDNA of all samples.

### Effect of genotype and experimental challenge on gene expression

Genotype and the interaction between genotype and sample subgroup were non-significant (*P =* 0.55 and 0.26, respectively) and removed from the model for relative expression of *CXCR1*. Relative expression of *CXCR1* was significantly higher in milk somatic cells from the infected and neighboring quarters 12 h after inoculation compared to milk somatic cells isolated 1 h before inoculation (both *P* < 0.01). Additionally, relative expression of *B2M*, *H2A*, *HPRT1*, *SDHA* and *YWHAZ* differed significantly between milk somatic cells from the infected quarters 12 h after inoculation and milk somatic cells isolated 1 h before inoculation (*P* < 0.05). Furthermore, relative expression of *B2M* and *YWHAZ* differed significantly between milk somatic cells from the neighboring quarters 12 h after inoculation and milk somatic cells isolated 1 h before inoculation. Relative expression of the three most stable genes (*ACTB*, *RPS15A* and *UBC*) did not differ significantly between the subgroups (Table 4).

**Table 4:** **Linear mixed models describing the difference in gene expression in milk somatic cells before and after experimental challenge**

**Gene**	**All quarters 1 h**		**Neighboring quarters**			**Infected quarters**		
	**before inoculation** ^**a**^		**12 h after inoculation**			**12 h after inoculation**		
	**(n = 15** ^**b**^ **)**		**(n = 8)**			**(n = 7** ^**b**^ **)**		
	**β**_**0**_^**c**^	**SE** ^**d**^	**β** ^**c**^	**SE** ^**d**^	***P*** **-value**	**β** ^**c**^	**SE** ^**d**^	***P*** **-value**
*ACTB*	0.27	0.07	-0.06	0.08	0.46	0.10	0.09	0.27
*B2M*	0.04	0.06	0.15	0.07	<0.05	0.41	0.07	<0.01
*CXCR1*	-1.30	0.15	0.94	0.26	<0.01	1.71	0.27	<0.01
*H2A*	-0.04	0.06	0.17	0.07	<0.05	-0.21	0.07	<0.01
*HPRT1*	-0.08	0.05	-0.03	0.07	0.66	-0.31	0.07	<0.01
*RPS15A*	-0.10	0.10	-0.07	0.12	0.59	-0.06	0.13	0.62
*SDHA*	-0.13	0.05	0.04	0.08	0.67	-0.22	0.09	<0.05
*UBC*	-0.17	0.05	0.13	0.08	0.10	-0.03	0.08	0.67
*YWHAZ*	-0.13	0.08	0.05	0.09	0.61	0.28	0.09	<0.01

^a^Two quarters of 8 dairy heifers were inoculated with PBS or *Staphylococcus chromogenes*.

^b^Two samples showed insufficient cDNA integrity and were therefore removed from the dataset.

^c^Regression coefficient.

^d^Standard error.

Heifer and quarter were added as random effect to correct for clustering of quarters within cows and two observations per quarter, respectively.

## Discussion

Gene expression analysis in experimentally infected and healthy quarters allows for identification of differentially expressed genes and pathways. Quantitative real-time PCR in milk somatic cells isolated prior to inoculation and at different stages of experimental infection enables a detailed follow-up of the host response. In this study, we analyzed associations between SNP *CXCR1*c.980A > G and *CXCR1* expression in milk somatic cells and studied the influence of an experimental intramammary challenge with *S. chromogenes* on expression of *CXCR1* and eight commonly used reference genes. Because IMI in one quarter can influence gene expression and immunity in neighboring quarters [17,18], we compared values before and after challenge rather than infected and non-infected quarters.

Compared to biopsies, milk somatic cells allow for easy resampling but yield less RNA, especially if the SCC is low [19]. Because of the low yield in some samples, we opted to isolate RNA from all milk somatic cells rather than a subpopulation (e.g. neutrophils). RNA integrity can influence RT-qPCR results but is not easy to asses [20]. Besides analyzing rRNA integrity of a representative sample using gel electrophoresis, we designed two four-primer PCR assays amplifying three fragments of *YWHAZ* and *CXCR1* to test cDNA integrity of all samples. The assays are based on the fact that if integrity is too low, amplification of large fragments of approximately 500 and 900 bp will be affected. Latter fragments are more than 4 times as long as the amplicon of the target gene in the qPCR (118 bp).

Polymorphism c.980A > G was not associated with *CXCR1* expression in milk somatic cells. Yet, relative *CXCR1* expression increased in milk somatic cells isolated from infected quarters which corresponds well with *in vitro* research showing increased *CXCR1* expression in blood neutrophils after *in vitro* LPS challenge [21]. To a lesser extent, transcription also increased in milk somatic cells from neighboring quarters. This might be due to cross-talk with the infected quarters or due to the inoculation of PBS. Although SCC increased little in the neighboring quarters, we cannot exclude a minimal inflammation caused by the insertion of the catheter, the PBS or both. The much higher increase in SCC and *CXCR1* expression in the infected compared to the neighboring quarters suggests inflammation in the infected quarters to be mainly due to experimental IMI.

The experimental challenge had a significant effect on the relative expression of 5 out of 8 candidate reference genes. Important to mention is that candidate reference genes were selected based on a stable expression in other studies [13-15]. Reference genes in experimental infection studies should be stably expressed and unaffected by IMI. Validation of the reference gene to normalize gene expression data is not always published [20]. Although normalization to a single reference gene can cause relative large errors [22], it is often practiced [17,23]. If the expression of this single reference gene is affected by IMI, certain genes might be falsely identified as up- or downregulated whereas truly up- or downregulated genes might be missed.

## Conclusion

In conclusion, *CXCR1* expression in milk somatic cells was not associated with SNP c.980A > G but was upregulated following experimental IMI with *S. chromogenes*. Additionally, differential expression was observed for candidate fererence genes *B2M*, *H2A*, *HPRT1*, *SDHA* and *YWHAZ*. The effect of intramammary challenge on expression of reference genes should be tested and reported in future studies on gene expression in milk somatic cells.
